# Supplementary material for: Modulation of JAK-STAT Signaling by LNK: A Forgotten Oncogenic Pathway in Hormone Receptor-Positive Breast Cancer
Source: Int J Mol Sci. 2023 Sep 30;24(19):14777. doi: 10.3390/ijms241914777 (PMC10573125; doi:10.3390/ijms241914777)
Supplement: Supplementary file 1 [file ijms-24-14777-s001.zip › ijms-2557283-supplementary.pdf]

Table S1. List of the 13 studies included in the combined analysis for mutations of the selected proteins in the cBioPortal for Cancer Genomics database.

No.	Study	Reference
[1]	Breast Invasive Carcinoma (British Columbia, Nature 2012)	Shah, S. P., Roth, A., Goya, R., Oloumi, A., Ha, G., Zhao, Y., Turashvili, G., Ding, J., Tse, K., Haffari, G., Bashashati, A., Prentice, L. M., Khattra, J., Burleigh, A., Yap, D., Bernard, V., McPherson, A., Shumansky, K., Crisan, A., Giuliany, R., … Aparicio, S. (2012). The clonal and mutational evolution spectrum of primary triple-negative breast cancers. *Nature*, *486*(7403), 395–399. https://doi.org/10.1038/nature10933
[2]	Breast Invasive Carcinoma (TCGA, Pan-Cancer Atlas)	The Cancer Genome Atlas Research Network., Weinstein, J., Collisson, E. *et al.* The Cancer Genome Atlas Pan-Cancer analysis project. *Nat Genet* **45**, 1113–1120 (2013). https://doi.org/10.1038/ng.2764
[3]	Metastatic Breast Cancer (INSERM, PLoS Med 2016)	Lefebvre, C., Bachelot, T., Filleron, T., Pedrero, M., Campone, M., Soria, J. C., Massard, C., Lévy, C., Arnedos, M., Lacroix-Triki, M., Garrabey, J., Boursin, Y., Deloger, M., Fu, Y., Commo, F., Scott, V., Lacroix, L., Dieci, M. V., Kamal, M., Diéras, V., … André, F. (2016). Mutational Profile of Metastatic Breast Cancers: A Retrospective Analysis. *PLoS medicine*, *13*(12), e1002201. https://doi.org/10.1371/journal.pmed.1002201
[4]	The Metastatic Breast Cancer Project (Archived, 2020)	www.mbcproject.org/data-release
[5]	Breast Cancer (MSK, Clinical Cancer Res 2020)	Pareja, F., Brown, D. N., Lee, J. Y., Da Cruz Paula, A., Selenica, P., Bi, R., Geyer, F. C., Gazzo, A., da Silva, E. M., Vahdatinia, M., Stylianou, A. A., Ferrando, L., Wen, H. Y., Hicks, J. B., Weigelt, B., & Reis-Filho, J. S. (2020). Whole-Exome Sequencing Analysis of the Progression from Non-Low-Grade Ductal Carcinoma *In Situ* to Invasive Ductal Carcinoma. *Clinical cancer research : an official journal of the American Association for Cancer Research*, *26*(14), 3682–3693. https://doi.org/10.1158/1078-0432.CCR-19-2563
[6]	Breast Cancer (SMC 2018)	Kan, Z., Ding, Y., Kim, J., Jung, H. H., Chung, W., Lal, S., Cho, S., Fernandez-Banet, J., Lee, S. K., Kim, S. W., Lee, J. E., Choi, Y. L., Deng, S., Kim, J. Y., Ahn, J. S., Sha, Y., Mu, X. J., Nam, J. Y., Im, Y. H., Lee, S., … Park, Y. H. (2018). Multi-omics profiling of younger Asian breast cancers reveals distinctive molecular signatures. *Nature communications*, *9*(1), 1725. https://doi.org/10.1038/s41467-018-04129-4
[7]	Breast Invasive Carcinoma (Broad, Nature 2012)	Banerji, S., Cibulskis, K., Rangel-Escareno, C., Brown, K. K., Carter, S. L., Frederick, A. M., Lawrence, M. S., Sivachenko, A. Y., Sougnez, C., Zou, L., Cortes, M. L., Fernandez-Lopez, J. C., Peng, S., Ardlie, K. G., Auclair, D., Bautista-Piña, V., Duke, F., Francis, J., Jung, J., Maffuz-Aziz, A., … Meyerson, M. (2012). Sequence analysis of mutations and translocations across breast cancer subtypes. *Nature*, *486*(7403), 405–409. https://doi.org/10.1038/nature11154
[8]	Breast Invasive Carcinoma (Sanger, Nature 2012)	Stephens, P. J., Tarpey, P. S., Davies, H., Van Loo, P., Greenman, C., Wedge, D. C., Nik-Zainal, S., Martin, S., Varela, I., Bignell, G. R., Yates, L. R., Papaemmanuil, E., Beare, D., Butler, A., Cheverton, A., Gamble, J., Hinton, J., Jia, M., Jayakumar, A., Jones, D., … Stratton, M. R. (2012). The landscape of cancer genes and mutational processes in breast cancer. *Nature*, *486*(7403), 400–404. https://doi.org/10.1038/nature11017
[9]	Proteogenomic landscape of breast cancer (CPTAC, Cell 2020)	Krug, K., Jaehnig, E. J., Satpathy, S., Blumenberg, L., Karpova, A., Anurag, M., Miles, G., Mertins, P., Geffen, Y., Tang, L. C., Heiman, D. I., Cao, S., Maruvka, Y. E., Lei, J. T., Huang, C., Kothadia, R. B., Colaprico, A., Birger, C., Wang, J., Dou, Y., … Clinical Proteomic Tumor Analysis Consortium (2020). Proteogenomic Landscape of Breast Cancer Tumorigenesis and Targeted Therapy. *Cell*, *183*(5), 1436–1456.e31. https://doi.org/10.1016/j.cell.2020.10.036
[10]	Metaplastic Breast Cancer (MSK,NPJ Breast Cancer 2021)	da Silva, E. M., Selenica, P., Vahdatinia, M., Pareja, F., Da Cruz Paula, A., Ferrando, L., Gazzo, A. M., Dopeso, H., Ross, D. S., Bakhteri, A., Riaz, N., Chandarlapaty, S., Razavi, P., Norton, L., Wen, H. Y., Brogi, E., Weigelt, B., Zhang, H., & Reis-Filho, J. S. (2021). TERT promoter hotspot mutations and gene amplification in metaplastic breast cancer. *NPJ breast cancer*, *7*(1), 43.

		https://doi.org/10.1038/s41523-021-00250-8
[11]	Breast Cancer (MSK, Cancer Cell 2018)	Razavi, P., Chang, M. T., Xu, G., Bandlamudi, C., Ross, D. S., Vasan, N., Cai, Y., Bielski, C. M., Donoghue, M. T. A., Jonsson, P., Penson, A., Shen, R., Pareja, F., Kundra, R., Middha, S., Cheng, M. L., Zehir, A., Kandoth, C., Patel, R., Huberman, K., … Baselga, J. (2018). The Genomic Landscape of Endocrine-Resistant Advanced Breast Cancers. *Cancer cell*, *34*(3), 427–438.e6. https://doi.org/10.1016/j.ccell.2018.08.008
[12]	Breast Cancer (MSK, Nature Cancer 2020)	Razavi, P., Dickler, M. N., Shah, P. D., Toy, W., Brown, D. N., Won, H. H., Li, B. T., Shen, R., Vasan, N., Modi, S., Jhaveri, K., Caravella, B. A., Patil, S., Selenica, P., Zamora, S., Cowan, A. M., Comen, E., Singh, A., Covey, A., Berger, M. F., … Chandarlapaty, S. (2020). Alterations in *PTEN* and *ESR1* promote clinical resistance to alpelisib plus aromatase inhibitors. *Nature cancer*, *1*(4), 382–393. https://doi.org/10.1038/s43018-020-0047-1
[13]	Breast Cancer (MSK, NPJ Breast Cancer 2019)	Nixon, M. J., Formisano, L., Mayer, I. A., Estrada, M. V., González-Ericsson, P. I., Isakoff, S. J., Forero-Torres, A., Won, H., Sanders, M. E., Solit, D. B., Berger, M. F., Cantley, L. C., Winer, E. P., Arteaga, C. L., & Balko, J. M. (2019). PIK3CA and MAP3K1 alterations imply luminal A status and are associated with clinical benefit from pan-PI3K inhibitor buparlisib and letrozole in ER+ metastatic breast cancer. *NPJ breast cancer*, *5*, 31. https://doi.org/10.1038/s41523-019-0126-6

Note: The above data was cited from cBioPortal for Cancer Genomics data base (http://www.cbioportal.org/index.do; 25 July 2023).
